# Candidate Performance and Observable Audience Response: Laughter and Applause–Cheering During the First 2016 Clinton–Trump Presidential Debate

**DOI:** 10.3389/fpsyg.2018.01182

**Published:** 2018-07-20

**Authors:** Patrick A. Stewart, Austin D. Eubanks, Reagan G. Dye, Zijian H. Gong, Erik P. Bucy, Robert H. Wicks, Scott Eidelman

**Affiliations:** ^1^Department of Political Science, University of Arkansas, Fayetteville, AR, United States; ^2^Department of Psychological Science, University of Arkansas, Fayetteville, AR, United States; ^3^College of Media and Communication, Texas Tech University, Lubbock, TX, United States; ^4^Department of Communication, University of Arkansas, Fayetteville, AR, United States

**Keywords:** defining moments, observed audience response (OAR), laughter, applause–cheering, booing, presidential debates, intra-audience effects

## Abstract

Raucous audience applause–cheering, laughter, and even booing by a passionately involved electorate marked the 2016 presidential debates from the start of the primary season. While the presence and intensity of these observable audience responses (OARs) can be expected from partisan primary debates, the amount of not just laughter, but also applause–cheering and booing during the first general election debate between Hillary Clinton and Donald Trump was unprecedented. Such norm-violating audience behavior raises questions concerning not just the presence, strength, and timing of these OAR, but also their influence on those watching on television, streaming video, or listening to radio. This report presents findings from three interconnected studies. Study 1 provides a baseline for analysis by systematically coding the studio audience response in terms of utterance type (laughter, applause–cheering, booing, and mixtures), when and how intensely it occurred, and in response to which candidate. Study 2 uses observational analysis of 362 undergraduate students at a large state university in the southern United States who watched the debate on seven different news networks in separate rooms and evaluated the candidates’ performance. Study 2 considered co-occurrence of OAR in the studio audience and in the field study rooms, finding laughter predominated and was more likely to co-occur than other OAR types. When standardized cumulative strength of room OAR was compared, findings suggest co-occurring OAR was stronger than that occurring solely in the field study rooms. Analysis of truncated data allowing for consideration of studio audience OAR intensity found that OAR intensity was not related to OAR type occurring in the field study rooms, but had a small effect on standardized cumulative strength. Study 3 considers the results of a continuous response measure (CRM) dial study in which 34 West Texas community members watched and rated the candidates during the first debate. Findings suggest that applause–cheering significantly influenced liking of the speaking candidate, whereas laughter did not. Further, response to applause–cheering was mediated by party identity, although not for laughter. Conclusions from these studies suggest laughter as being more stereotypic and likely to be mimicked whereas applause–cheering may be more socially contagious.

## Introduction

The 2016 election can be seen as one in which a passionately involved electorate was key for its unexpected outcome as novice political outsider Donald Trump became president of the United States. Trump’s success defied early predictions, with few political experts anticipating the intensity from his base of support when compared to more traditional candidates during both the Republican primaries and general election. Despite dispensing with traditional expectations and violating presidential debate norms, Trump’s performance and the associated audience response of raucous applause–cheering, laughter, and even booing during the initial 2016 primary debates (Stewart et al., 2016) and the general election debates can be seen as providing insights concerning his populist appeal. Beyond their populist overtones, these observable audience responses (OARs) can thus be seen as valid and reliable audible indicators of the intensity of shared individual and emergent group attitudes toward political candidates more generally (Stewart, 2012, 2015; Stewart et al., 2016).

Existing debate-focused research has documented the role of these salient media events in reinforcing existing preferences, producing issue knowledge, and influencing perceptions of candidate character, thus affecting undecided voter choices (McKinney and Warner, 2013). Debate viewing may also reorder the relative importance of issues in viewers’ minds and shift leadership potential to the foreground as a salient consideration (Benoit et al., 2001; Schrott and Lanoue, 2008; Schroeder, 2016). However, most existing research treats debates as monolithic events and examines overall debate effects rather than communication dynamics occurring during the debates themselves.

While providing useful insights concerning the impact of mediated events on electoral dynamics, these approaches do not take into consideration the unpredictable events that occur during debates and how they affect perceptions. Even after accounting for how campaigns pitch-and-spin their candidates’ performance (Norton and Goethals, 2004; Schroeder, 2016) multiple, relatively unexplored factors occurring during the debate affects candidate evaluations. Candidate rhetorical approach (Benoit, 2013), non-verbal behavior (Bucy and Stewart, Forthcoming), and media presentation style (Cavari et al., 2017) influence debate viewer perceptions. In other words, most research does not consider the process of change in debate viewer perceptions or those critical defining moments, which are often met with audience laughter, applause–cheering, and/or booing (Clayman, 1995).

Recent research addresses this oversight through continuous response measures (CRMs) and dial testing of debates, eye tracking of candidate exchanges, and focus group analysis of memorable debate moments (Gong and Bucy, 2015). Analysis of social media such as Twitter also suggests that candidate non-verbal behavior, even more so than their verbal acclaims, attacks and defenses (Shah et al., 2015), influence audience response. Still, these approaches may not capture the contemporaneous, in-person emotional response of viewers, instead representing more considered appraisals (Nagel et al., 2012) prone to social conformity pressures (Fein et al., 2007; Davis et al., 2011). Furthermore, by focusing on individual response such measures might be missing the highly important attribute of implicit sociality embedded with audible responses by the audience, especially during emotionally charged political events.

Research considering OAR to political candidates at events such as debates tends not to focus on the audience itself, and its social influence on other audience members. The existing research that does consider OAR on participant evaluation, including those considering political figures, are experimental and do not disambiguate positive response such as laughter, applause, and/or visually oriented non-verbal signals (Hylton, 1971; Duck and Baggaley, 1975; Cummins and Gong, 2017). Specifically, the studies by Wiegman (1987) and Fein et al. (2007), while providing insight into the social influence of OAR on participant evaluation of the candidates and policy issues, tend to include both audible reactions and OAR. For instance, Wiegman (1987) carried out a field experiment with a well-known Dutch political figure that involved a studio audience either reacting positively, negatively, or neutrally through a range of audible utterances and variety of gestures and facial displays. Fein et al. (2007) found that “… absent the applause, laughter, and general approval of [United States President Ronald] Reagan’s one-liners, these responses were not seen as particularly noteworthy by the participants.” (p. 178) However, they did not differentiate between applause and laughter nor the moderator’s verbal and non-verbal response.

While not dealing with political figures, Axsom et al. (1987) auditory-based lab experiment comparing “enthusiastic applause–cheering” to unenthusiastic and polite applause with occasional derisive cries, found that OAR influenced response to a specific policy issue (imprisonment vs. probation). They noted that “the persuasive impact of audience cues may reflect subjects’ tendencies to use a simple consensus heuristic such as “if other people think the message is correct, then it is probably valid.” (p. 39) In summary, while previous research provides useful insights, a gap in the literature exists by the authors not differentiating between OAR types or systematically considering OAR intensity.

The existing research that does differentiate between the types of OAR tends to consider these utterances as a means by which large groups of followers provide feedback to their leaders. Specifically, audience responses such as applause–cheering, laughter, and booing provide audible signals indicating the type of response while indexing level of follower support or opposition. Furthermore, the timing of OAR indicates their level of synchrony with the speaker, as well as that with fellow audience members (Bull and Wells, 2002). Thus, the type and magnitude of the OAR supplies audible information indicating coalition size and strength (Dunbar, 1993) providing the speaker with immediate and unobtrusive feedback that may be continuously monitored and allow for enhanced speechmaking (West, 1984).

At the same time, media audiences, whether streaming the debates, watching on television, or listening through other broadcast media, as well as journalists reporting on the event, may be affected by this information. Indeed, OAR can lead to change regarding how the speaker is evaluated, indeed, even more so than the eliciting comments themselves (Fein et al., 2007). In other words, social influence asserted through OAR affects resultant viewer and listener perceptions, attitudes, and behavior; however, the specific influence of different OAR such as applause–cheering, laughter, and booing remains to be studied in depth.

### Observable Audience Response (OAR) Reliability

Observable audience response such as applause–cheering, laughter, and booing may be seen as belonging to a class of behavior that is almost automatic and highly contagious, which in turn might lead to affective, cognitive, and behavioral response with political implications (Fein et al., 2007). In other words, there likely is a high level of behavioral mimicry by audience members as they match each other’s audible response (Sachisthal et al., 2016; Moody et al., 2017). This audible response may in turn influence individual emotional response, and with it the evaluation of the candidate eliciting the response (as well as those sharing in the response) through emotional contagion (Hatfield et al., 1994, 2014; Lakin et al., 2003). These group vocalizations can thus provide evidence of the type and intensity of connection the audience members have with the candidates, and perhaps as important, the members have with each other in the room.

The overarching issue regarding OAR concerns their reliability in differentially reflecting the audience’s putative emotional and behavioral intent. Here, reliable indicators of emotion may be defined as being first, an accurate recognition of the emotional state of the communicator, and their resultant behavioral intent, and second, the signal being an index of the sender’s underlying state by being costly to produce (Mehu et al., 2011). Because of the social nature of group vocalizations, these utterances should be stereotyped and contagious; in other words, such behaviors as laughing and yawning have coherent and identifiable vocalic, facial and even postural display behavior associated with them. As defined by Hatfield et al. (2014) this primitive social contagion is “(T)he tendency to automatically mimic and synchronize facial expressions, vocalizations, postures, and movements with those of another person’s and, consequently, to converge emotionally” (p. 169).

Despite the rather sparse nature of existing research on location of debate viewing and audience composition, we expect differences in the in-person studio audiences and those having a mediated experience. In other words, the studio audience likely reacts differently from those watching a video of the event. This may be due in part to a location’s acoustic qualities that may enhance or diminish the subjective emotional and physiological response of audience members (Stewart, 2012; Pätynen and Lokki, 2016) as well as the physical presence of contending candidates. Differences in response may further be affected by whether individuals are watching independently or amongst other individuals, whether known acquaintances or strangers, with increased laughter, if not the other OAR types, occurring with greater sociality. Furthermore, social norms likewise play a role in what is acceptable behavior or not, although this may be determined by audience member assumptions and relationships with each other (Devereux and Ginsburg, 2001; Platow et al., 2005; Fridlund, 2017).

Thus, in addition to the type of OAR (e.g., applause–cheering, laughter, and booing) identified and potential mixtures that might occur, the intensity of studio audience response may be characterized by its length in time combined with its perceived audible strength. This intensity may in turn affect onlookers, whether in the studio audience – yet not affiliated with any social group or faction – or watching on television, live streaming over the internet, or listening on the radio and thus experiencing intra-audience mediated effects from the OAR (Cummins and Gong, 2017).

Research Question 1: Is there a relationship between the presence of television studio audience OAR and the field study audience OAR?

Research Question 2: Is there a relationship between the intensity of television studio audience OAR and the strength of the field study audience OAR?

### Observable Audience Response (OAR) Types: Laughter, Applause–Cheering, and Booing

Generally speaking, one can identify three general types of audible OAR as applause–cheering^1^, laughter, and booing each serve to signal shared audience response to political candidates (Atkinson, 1984; West, 1984; Heritage and Greatbatch, 1986; Clayman, 1992, 1993; Bull and Miskinis, 2014). These OAR types, in addition to their effects being characterized by length and strength, may be accentuated or attenuated depending on audience member characteristics and the intensity of their response. Each OAR type serves distinct communicative ends allowing for audiences to communicate their support or disapproval for statements by leaders and putative leaders, with concomitant intensity and mixtures providing insight concerning passion and unanimity regarding these positions.

Laughter is the most studied of all vocalizations discussed here; however, the focus tends not to be on the group. Individual laughter is focused on due to it serving as a pervasive social signal in interpersonal interactions by punctuating speech and indicating speaking turn taking and transition (Provine, 1993; Gilmartin et al., 2013; Bonin et al., 2014; Scott et al., 2014). Individual laughter can indicate social intent through it being voiced and unvoiced (Bachorowski and Owren, 2001; Owren and Bachorowski, 2003) as well as communicating the different emotions of amusement, contempt, schadenfreude, and tickle (Szameitat et al., 2009, 2011).

As a result, laughter may be seen as a costly signal by virtue of it either being evoked in a manner that is difficult to control whereas even emitted laughter that is initially faked leads to physiological change (Provine, 1992; Bachorowski and Owren, 2001; Devereux and Ginsburg, 2001; Ruch and Ekman, 2001; McGettigan et al., 2015). Individual laughter likewise serves as a social lubricant by affecting subject mood states by decreasing negative affect, increasing positive affect and enhancing pain tolerance while increasing social cooperation and group identity (Van Vugt et al., 2014). It thus serves as a highly reliable social signal regarding behavioral intent (van Hooff and Preushoft, 2003; Panksepp, 2007; Pellis et al., 2014).

When considering group level behavior, research regarding laughter tends to focus on the target and intent of the verbal utterances leading to this type of response (Wells and Bull, 2007; Stewart, 2012; Choi et al., 2016). Thus, research concerning group laughter tends to reflect findings regarding response to individual speakers. The group vocalic utterances of laughter is limited in length of time to a much greater extent than those created through rhythmic mechanical noisemaking such as applause tending to last from 1 to 3 s in comparison with 2 to 8 s for applause–cheering (Stewart, 2015), as well as likely booing (although these types of rare OAR makes strong assertions untenable). Furthermore, when an audience shows their appreciation for a humorous comment, applause–cheering prolongs the laughing utterance (Stewart, 2012; Stewart et al., 2016). This suggests high levels of social mimicry in the immediate OAR and then likely social contagiousness through its continuation.

Of all the forms of OAR, applause–cheering is perhaps most likely to be observed in group settings such as political speeches and intra-party debates. This is likely due to the ease with which candidates are able to evoke it among supporters in partisan settings. As a result, applause–cheering has been appreciated for the role it plays in providing an important barometer of a politicians’ individual appeal during speeches (Atkinson, 1984; West, 1984; Heritage and Greatbatch, 1986; Bull, 2003) or when in direct competition with other candidates during debates (Stewart, 2015; Stewart et al., 2016).

On the other hand, due to applause–cheering likely not being as costly to produce physiologically and easier for audience members to inhibit than laughter (Stewart, 2015), it might not be as reliable a social signal. That does not mean that this activity is not stereotyped and thus easy to identify while also being contagious. Research concerning applause bouts in small groups (13–20) found that most involve only 9–15 claps per person, although some last over 30 claps (Mann et al., 2013). A study considering applause in larger groups suggests this activity typically begins with an uncoordinated loud burst of high frequency clapping that then synchronizes through a form of social contagion and coordination (Néda et al., 2000a,b). Thus, while the initial applause is louder, the synchronicity of OAR afterward suggests social contagion between audience members.

Much rarer than supportive in-person audience response through laughter and applause–cheering at political events are boos and jeers (Clayman, 1992, 1993; Bull and Miskinis, 2014). However, besides research regarding individuals jeering/heckling carried out over 40 years ago (Sloan et al., 1974; Silverthorne and Mazmanian, 1975), little research on the nature of booing, especially regarding physiological characteristics and group-level attributes, has been carried out. Existing research on booing finds it rarely occurring. Even in the highly divisive 2016 presidential primary debates, booing, both alone and mixed with applause–cheering and laughter, occurred only in 5% of OAR observed (Stewart et al., 2016). Beyond audibly signaling negative response, the intent and target matters; disaffiliative booing by the “right” crowd can enhance electoral status by emphasizing willingness to take an unpopular stand whereas affiliative booing may be used to attack on out-group leaders and policy positions (Bull and Miskinis, 2014). However, the key factor is that the booing occurred during speeches in front of relatively coherent partisan audiences.

In summary, laughter, applause–cheering, and booing provide means by which the audience physically present with a politician can communicate as a group in distinctive and easily identifiable ways. While pre-verbal, these OAR can successfully be used to strategically communicate factional preferences to not just the speaker, but also to other potential group members. As a result, there are social benefits and costs from participating or not participating in OAR; audience members must consider if engaging in different OAR types will be socially costly to them or if joining in with other audience members when candidates break norms of politeness and civility will pay off socially (Dailey et al., 2005). To the point, the social norms of politeness by audience members instructed to not influence the proceedings through their laughter, applause–cheering, and booing can be contravened if their preferred candidate welcomes, even incites it, and there is no effective sanction laid upon them. While we expect the candidates to successfully evoke OAR through punchlines, claptrap, and all manner of rhetorical tools at their disposal, the type of OAR will likely vary systematically. Because laughter is difficult to control, we do not expect that the candidate evoking it will influence either its occurrence or the strength of the field study audiences’ response. On the other hand, with applause–cheering we do expect that both the candidate making the comment inciting this response and the intensity of the studio audience’s response will influence the strength of the response.

Research Question 3: Is there a relationship between studio audience OAR type and field study audience OAR type?

Research Question 4: Is there a relationship between studio audience OAR type and field study audience OAR intensity?

### Present Research

This report presents the findings of distinct, yet interconnected studies to explore the nature of OAR and their potential influence on evaluation of presidential candidates during a general election debate. We take a bottom-up/reverse engineering approach to study behavior as it occurs in a naturalistic environment (de Gelder, 2017); essentially we use the observational methods used in human ethology (Schubert, 1988; Eibl-Eibesfeldt, 1989; Masters, 1989; Weisfeld, 1993; Salter, 2007) and apply them to a political event of great importance as it occurs. As a result, this report is by necessity correlational and exploratory.

We focus on the first general election debate between Donald Trump and Hillary Clinton in a multipart approach. Study 1 uses ANVIL content coding software to characterize and analyze studio audience response in terms of when the OAR occurred, what type they were (laughter, applause–cheering, booing, and mixtures), their duration and perceived strength, and in response to which candidate. Study 2 builds off of Study 1 by collecting and analyzing a unique dataset in which 362 undergraduate students took part in a field experiment watching or listening to the first presidential general election debate in seven different rooms. We use ethological analysis of the field study participants’ OAR by considering when different types occurred and how strong they were perceived to be by observers. This allows us to compare relatively unfettered field study audiences to the studio audience, where moderator instructions and politeness expectations presumably played a role in constraining an elite partisan audience, to the less inhibited university student-occupied rooms. We draw conclusions regarding both laughter and applause–cheering by considering four research questions concerning the co-occurrence of the OAR of laughter and applause–cheering (i.e., simultaneously occurring in both the studio audience and in the field study rooms). With Study 3, we evaluate the effect of studio audience laughter and applause–cheering on mediated viewer moment-to-moment (MTM) response of liking the speaking candidate. We finish this report by discussing the implications of our findings for future research.

## Study 1

### Materials and Methods

The first of three general election debates between Democratic Party presidential nominee Hillary Clinton and Republican Party nominee Donald Trump occurred the evening of Monday September 26, 2016 and was hosted outside New York City by Hofstra University. Sponsored by the *Commission on Presidential Debates* and moderated by NBC News anchor Lester Holt, the 90-min debate focused on the topics *achieving prosperity*, *America’s direction*, and *securing America* with specific questions regarding jobs, race relations, taxes, and the prospect of cyberattacks. With an estimated 84 million viewers, the highly anticipated first head-to-head confrontation between Trump and Clinton became the most watched debate in United States history (Cavari et al., 2017).

Speaking time and studio audience OAR used ANVIL content analysis software, which allows for frame-by-frame coding (Kipp, 2012). The inter-coder reliability (ICR) between two coders considering speaking time and OAR assessed approximately 30 min randomly chosen from video clips coded surpassed acceptable levels (κ > 0.92).

### Findings

Trump had nearly 5 min more speaking time at 47 min (2,795 s) when compared to Clinton’s 42 min (2,492 s). This was likely due to his interruptions, as Trump had nearly twice as many speaking turns (*n* = 80) as Clinton (*n* = 43). With moderator Lester Holt’s speaking time of 10 min (597 s) and 91 speaking turns, the total floor time of the three debate participants was 98 min over 214 total speaking turns, suggesting a high level of overlap.

A total of 34 OAR were identified during the debate proper (we did not code for the welcoming or concluding applause). These 34 studio audience OAR to the candidates’ statements/retorts – or in one case response to the moderator – lasted a total of 102.72 s and averaged just over 3 s (*M* = 3.02; *SD* = 1.96). When considering types of OAR, 21 laughter (*M* = 2.09; *SD* = 1.20; Min = 0.4, Max = 4.17), nine applause–cheering (*M* = 5.48; *SD* = 1.48; Min = 3.4, Max = 7.97), two booing (*M* = 1.52; *SD* = 0.26; Min = 1.33, Max = 1.7), and two mixed vocalizations [applause and laughter (4.3 s); applause and booing (2.17 s)] were identified. Due to the lack of variance, the two booing and two mixed responses are omitted from statistical analyses, but considered in the descriptive analysis.

In addition to evaluating length of the audience’s utterances, we coded for the subjective strength of these responses on a 1- to 5-point scale ranging from “barely audible” to “extremely audible” (Ekman and Friesen, 2003) using three coders (α = 0.76). The mean of the three was computed to form our *strength* variable (*M* = 2.78; *SD* = 1.26). Due to the high level of correlation between these two measures of OAR length and strength (Pearson’s *r* = 0.81), we created an additive studio audience intensity index (*M* = 5.08; *SD* = 3.06). Throughout this manuscript we report *t-* and *p-*values to allow for standard statistical consideration, but note that analysis of the population of studio audience and field study OAR means that such statistical standards are not strictly appropriate.

### Discussion

In comparison with previous general election debates (Rhea, 2012; Stewart, 2012) the first 2016 meeting between Hillary Clinton and Donald Trump was a raucous affair just in terms of the 21 laughter events. This finding aligns with expectations and findings suggesting that while both OAR types involve levels of social contagion, laughter likely is more reliable due to the relative absence of control over it (Stewart, 2015). However, it is the amount of voluntary audience involvement that sets this debate apart. Nine (26.5%) OAR involved applause and cheering, one involved laughter mixed with applause, and two involved booing, with a third occurrence of booing in conjunction with laughter.

**FIGURE 1:**
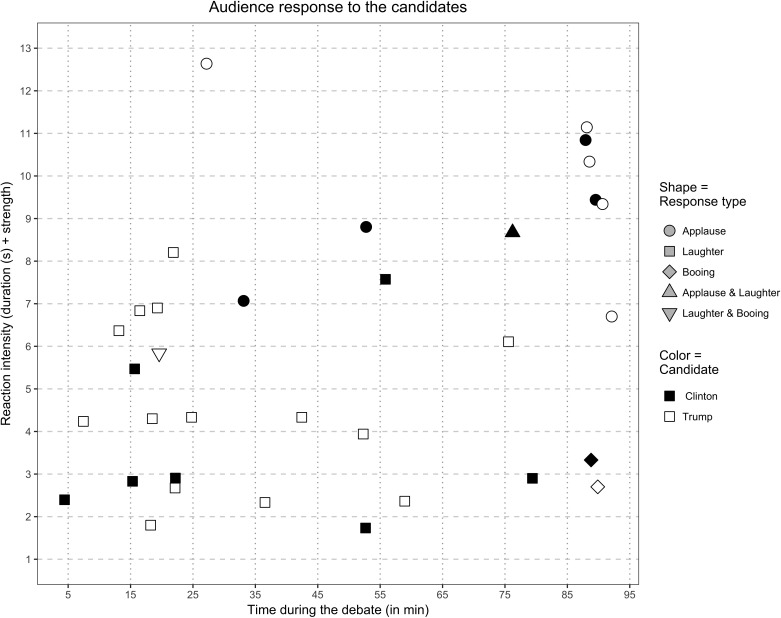
Studio audience OAR to Hillary Clinton and Donald Trump during first 2016 presidential debate.

The norms of civility respected in previous presidential debates by the audience through their OAR were not followed in the first 2016 general election presidential debate. Arguably, the norm-bending behavior of Trump through his many interruptions and perhaps more importantly, his use of laughter-inducing rhetoric led to the studio audience departing from customary expectations concerning their collective behavior (Dailey et al., 2005). This is not to diminish Clinton’s or moderator Lester Holt’s role in audience actions. Clinton’s attacks on Trump likely stirred a defensive group response from his supporters. When **Figure 1** is considered, Holt’s lack of control over the audience can be seen with escalating incidence and intensity of laughter. This likely enabled the more consciously controlled studio audience applause–cheers in response to Trump’s attack on Clinton’s email controversy to occur.

The ability of both candidates to instigate OAR suggests similarities; however, there are revealing differences. Specifically, while both Trump and Clinton invited equal numbers of studio audience applause–cheering with four apiece, Trump was able to elicit five more studio audience OAR than Clinton. This was mainly through his laughter-eliciting attacks; he was also arguably more polarizing by eliciting boos-jeering in one case and a combination of laughter and boos in another instance. For her part, Clinton produced laughter followed by cheers in two cases, suggesting unconstrained support by her followers, especially in response to her attacks on Trump.

## Study 2

### Materials and Methods

Questions remain concerning the nature of the relationship between OAR by those in the studio audience and those watching the presidential debate on television, streaming on the internet, or listening on radio. Individuals hearing studio audience OAR in response to candidates utterances may potentially have also have experience intra-audience mediated effects through the OAR (Cummins and Gong, 2017) and been affected not just by the candidate statements (Fein et al., 2007). This intra-audience effect had the potential to affect millions, especially undecided voters, and more explicitly sets the stage for testing Research Questions 1–4.

### Participants and Method

To understand the potential influence of both the candidate utterances and studio audience response on network viewers observing the televised debate, this study built from a field experiment being conducted at large university in the southern United States (Cavari et al., 2017). Participants were recruited from approximately 2,000 undergraduate students in more than 100 communication, political science, and psychology course sections from the researchers’ home departments, received extra credit for taking part, and were not informed as to the study’s purpose.

A total of 610 participants filled out an online omnibus survey prior to the debate (between August 29 and September 26, 2016) and were randomly assigned to one of seven rooms after their identity was verified. Each room, which was built to hold from 46 to 138 individuals, presented a different network (*ABC, FOX News, MSNBC/NBC, CNN, NPR, CBS, C-SPAN*) to 42–57 participants in classrooms. Post-test survey data was collected immediately after the debate, but due to the unanticipated amount of OAR, was not usable due the ceiling effect on pertinent measures.

The debate was viewed by 362 participants who took part as specified by university IRB protocols. Usable post-debate data from the 341 participants who filled out and returned the post-debate survey showed the sample was composed of 64% females, had a mean age of 19.53 (*SD* = 2.71) and was predominantly Caucasian (83%; African American [6%], Hispanic [4%], Asian [3%], Native American [1%], the remainder self-identified as “other”).

Politically approximately 77% reported being registered voters, half (50.1%) self-identified as Republicans, just over a quarter (27.6%) as Democrats, and the remainder as independent/non-affiliated (22%). Political ideology as measured on a 7-point Likert-type scale (1 = *very liberal;* 7 = *very conservative*) was normally distributed and slightly right of center (*M* = 4.30). Chi-square (*p_gender_* = 0.59; *p_party_* = 0.29; *p_race_* = 0.85) and ANOVA (*p_ideology_* = 0.15; *p_age_* = 0.16) analyses show no significant room differences suggesting successful random assignment.

The participants were observed in seven different on-campus classrooms by study volunteers drawn from University Honors students and graduate students in Communication, Political Science, and Psychological Science programs. All rooms had three observers positioned at both front and one back room corners except the room watching ABC, which had six observers due to the additional observers mistakenly reporting to the incorrect room. Additionally, one observer was removed from analysis for coding only two OAR, when the average was 28.13 (*SD* = 8.65). In addition to checking in the students and keeping order, these observers were instructed to identify and code the field study room OAR in terms of type (Applause/Clapping, Laughter, Booing/Jeers, Other response), time that it occurred (to the minute), the individual (Clinton, Trump, or moderator) eliciting the OAR, the perceived strength of the OAR (see Study 1), and a brief description of the evoking comment or action.

To analyze the co-occurrence of field study room OAR with studio audience OAR, in other words the intra audience media effects, data for each of the field study rooms were first considered in terms of what was being observed and measured before being aggregated for analysis. Thus, we initially consider how OAR is not necessarily experienced and coded in the same manner. First, an OAR may be experienced and coded as having greater strength due to the observers’ proximity to the individual(s)’ utterance, and not necessarily due to the entire room vocalizing at higher levels. Second, identification of OAR type, whether laughter, applause–cheering, booing, or combinations of these, may be influenced by the strength of the OAR itself. In either case, greater involvement from greater numbers of audience members might lead to either enhanced clarity of signal, or greater ambiguity.

### Findings

**FIGURE 2:**
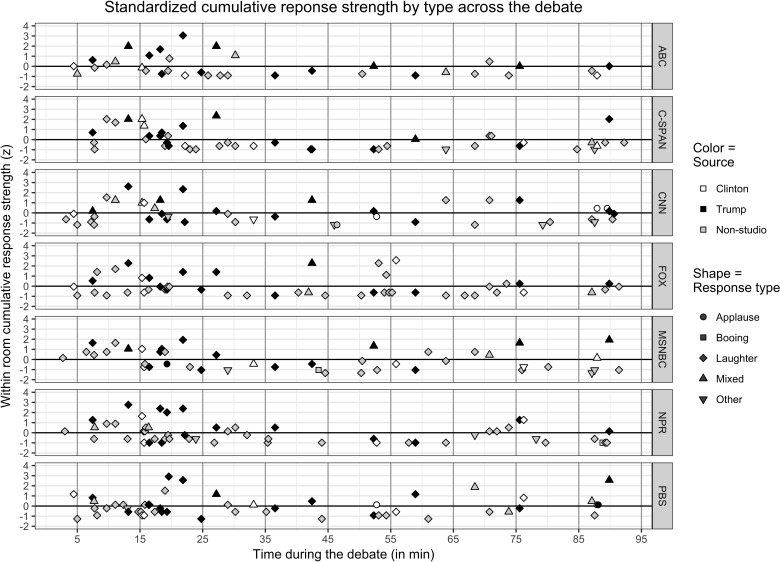
Field study OAR to Hillary Clinton and Donald Trump during first 2016 presidential debate.

Based upon the time of the occurrence and the comments, we were able to identify 113 unique OAR across the seven field study rooms with all showing a similar pattern (**Figure 2**), including which responses co-occurred with the studio audience (as noted in Study 1). While each of the field study rooms had multiple OAR that did not co-occur with those by the studio audience, we focused on those that represent a co-occurrence of audience response potentially signifying either shared response to candidate utterances or social contagion.

From this data, a clear pattern of agreement emerges: of the 321 verified field study room OAR correlating with candidate or moderator utterances, nearly four-fifths (*n* = 255; 79.4%) involved exclusively laughter. Of the other OAR, only 10 (3.1%) were distinguishable as solely applause–cheering (*n* = 8) or booing (*n* = 2). The remaining room responses were either identified as a mixture of applause–cheering and laughter (*n* = 1), laughter and booing (*n* = 3), an unidentified mixture (*n* = 41) or as no selection/other (*n* = 11). Thus we aggregated these responses into an “other” category.

While it is apparent that laughter predominated and was the most easily identified of OAR, with from one-to-three coders (or in the case of the ABC room, one-to-six coders) in each room, the level of agreement does not necessarily reflect ICR so much as the location of the coder and the individual(s) audibly responding to the debate and the strength of the OAR itself. For instance, while when laughter occurred there was strong inter-observer agreement, the other types of OAR rarely resulted in agreement. There may be a notable relationship between the observers distinctively hearing laughter, applause, booing, or mixtures of these responses due to position in the room. Some coders may perceive one type of OAR as more prominent due to proximity to the audible response within the field study room. As such, inter-coder approaches typically used with content analysis (e.g., Cronbach’s alpha, Krippendorff’s alpha) are not appropriate; instead, we develop a variable of cumulative strength. Cumulative strength thus considers the OAR occurring in each room and creates an index where each of the observers, using the 1–5 strength scale used in Studies 1 and 2, add their scores together. Next, due to the disparity in the number of coders across all rooms, cumulative strength was standardized within each respective room by creating z-scores allowing us to compare across treatment rooms.

#### Co-occurrence of Studio and Field Study OAR: Full Sample

In keeping with the previous studies, and due to statistical reasons, we do not consider co-occurrence of studio audience and field study audiences deriving from studio audience applause-and-laughter (*n* = 5 rooms), laughter-and-booing (*n* = 2 rooms), or booing (*n* = 8 rooms). This leaves us with a total of 306 field study OAR in the seven rooms characterized based upon type of OAR (laughter = 244; mixed = 62) and cumulative strength. This allows us to consider the influence of studio audience OAR intensity and type (laughter = 109; applause = 22; no response = 175) on field study room OAR.

When this categorical data is analyzed we find a highly significant relationship between types of studio audience and field study OAR co-occurring, χ^2^(2,306) = 27.790, *p* < 0.001, with a moderately strong relationship (Cramer’s *V* = 0.301). Specifically, marginally more field study audience laughter was observed in the seven rooms than was expected when studio audience laughter occurred (5.1) or there was no studio audience OAR (4.5). However, there were substantially fewer laughter responses in the field study room when there was studio audience applause (−9.5).

To assess the effect of the studio audience OAR on cumulative strength of field study OAR, we ran 3 (type of studio audience OAR: laughter, other, no response) × 7 (field study room) ANOVA on cumulative strength of OAR in field study rooms. Findings suggest the difference in the type of OAR was highly significant and had a strong effect [*F*(2,285) = 28.904, *p* < 0.001, $\eta_{\text{p}}^{2}$ = 0.806]. Neither the field study room [*F*(6,285) = 0.535, *p* = 0.780, $\eta_{\text{p}}^{2}$ = 0.050] nor was the interaction between OAR and field study room [*F*(12,285) = 0.570, *p* = 0.865, $\eta_{\text{p}}^{2}$ = 0.023] significant.

*Post hoc* analysis of the effect of the different types of OAR (applause–cheering vs. laughter) on the standardized cumulative strength of response in the field study rooms found that studio audience applause–cheering (*p* < 0.01; *M* = 0.352, *SD* = 0.233) and laughter (*p* < 0.001; *M* = 0.352, *SD* = 0.090) was significantly stronger than when there was no studio audience OAR (*M* = -0.295, *SD* = 0.072). At the same time, there was no difference between applause–cheering and laughter (*p* = ns).

#### Co-occurrence of Studio and Field Study OAR: Truncated Sample

Finally, to assess the influence of the intensity of the studio audience OAR on field study room OAR we considered only those cases in which there was a co-occurrence of studio audience and field study OAR. This leaves us with a truncated sample of 131 events. To consider the effect of the studio audience OAR type and intensity on the field study’s OAR type and the standardized cumulative strength, we carried out a binary logistic regression and an ANCOVA, respectively. Both equations include the studio audience OAR intensity index as a covariate with the type of studio audience OAR (laughter or other) as a between-subjects factor.

The binary logistic regression analysis considered the field study audience rooms laughter or other OAR type was predicted by studio audience laughter or applause and the intensity of their response. The full model was significant χ^2^(1) = 20.495, *p* < 0.001, and moderately strong (Cox and Snell *R^2^* = 0.145 and Nagelkerke *R^2^* = 0.218). Analysis of the variables suggest that while the intensity index was not significant, Wald χ^2^ = 0.347, *p* = 0.556, studio audience OAR type was significant Wald χ^2^ = 9.285, *p* < 0.01. Studio audience OAR predicted field study laughter correctly 92% of the time (92/100) and other types of response 45.2% (14/31).

Analysis of the effect of studio audience OAR type and intensity on field study room OAR standardized cumulative strength, on the other hand, suggest both variables have influence. Findings show the studio audience OAR intensity index was significant, had a small effect, and was positively related to field study OAR (*F* = 18.179, *p* < 0.001, $\eta_{\text{p}}^{2}$ = 0.124). The effect of the studio audience OAR type was likewise significant and had a small effect (*F* = 12.117, *p* < 0.01, $\eta_{\text{p}}^{2}$ = 0.086) with studio audience OAR laughter (*M* = 0.343, *SD* = 1.128) having a stronger influence than applause–cheering (*M* = 0.224, *SD* = 0.873).

### Discussion

Despite taking a conservative approach regarding our analysis of co-occurring studio and field study OAR by not including those studio audience events where applause–cheering followed and combined with laughter, our findings indicate laughter was more evident in the field study rooms than in the studio audience. When co-occurring with studio audience OAR, there was a moderately strong relationship between the type of studio audience OAR (laughter or applause)/non-response and the field study audiences OAR type, with applause–cheering significantly less likely to co-occur with laughter.

Furthermore, the more stereotypical signaling nature of laughter, when compared with other types of OAR, is apparent even when taking into account the “success” of candidate utterances (as indexed through studio audience audible intensity). This may be seen as indicating laughter, even when aggregated in OAR, being more automatic and stereotyped when compared with all other responses, even when considering observational judgments.

While the findings are illuminating, it should be noted that younger audiences such as studied here will likely laugh more due to social pressures, such as the implicit lack of knowledge concerning the status/rank of those around them (Mehu and Dunbar, 2008; Mehu, 2011). Younger individuals might be more likely to behaviorally mimic others (Sachisthal et al., 2016; Moody et al., 2017), especially if they appraise themselves as belonging to the implicit in-group (Platow et al., 2005; Sachisthal et al., 2016). As can be seen in **Figure 2**, the greatest amount of laughter, both concurrently with the studio audience and independent of them, occurred across all seven field study rooms after 5 min of relative quiet and appeared to be clustered in the first 20–25 min of the debate. In this case, participants likely signaled themselves as belonging to the peer group as a fellow student by laughing (relatively) early and often. While student participants might be more likely to mimic others around them, they do not necessarily experience the emotional contagion resulting in attitudinal change toward the candidates. To assess this, Study 3 considers the influence of studio audience OAR on how well individuals like the candidates.

## Study 3

### Methods

Participants were recruited from a west Texas community as part of an election study announced on the local newspaper’s website. Due to continuous response theater using dedicated wireless dials, sample size was limited to 34 participants—the maximum number the room could accommodate during the debate. Partisan identification was divided between 14 Republican Party identifiers, 11 Independents, and 9 Democratic Party identifiers. Participants received a small monetary inducement in exchange for their participation. Age ranged from 18 to 73 (*M* = 36.60, *SD* = 17.88) with a slight majority of participants (*n* = 19, 54.3%) male.

The dependent variable, *candidate evaluation*, was derived from participants’ moment-to-moment (MTM) response to the speaking candidate using the DialSmith Perception Analyzer 8.0 through wireless handheld response dials. When watching the debate, participants used their dial to indicate their agreement to the statement, “I like the candidate who is speaking,” with response options ranging from 0 (*Strongly Disagree*) to 100 (*Strongly Agree*). Prior to the debate beginning, participants were asked to set their dials to the scale’s mid-point of 50.

To calculate participant response to studio audience laughter and applause–cheering during the debate, the MTM responses 10 s prior to the onset of studio audience OAR provided a baseline average from which deviations up to 5 s afterward were considered. Thus, positive MTM change scores represent a more favorable attitude toward the candidate. The first 5 s after the onset of OAR was analyzed in order to account for potential delayed MTM reaction to OAR, as well as the average duration of OAR lasting roughly 2–3 s.

Nineteen studio audience OAR comprised of laughter and 11 of applause–cheering identified in Study 1 are considered, with overlapping or indistinct OAR removed from analysis. Of these, nine studio audience laughter segments and five applause–cheering OAR occurred during or after Hillary Clinton’s comments, while 10 laughter and 6 applause–cheering OAR occurred during or after Donald Trump’s comments.

### Findings

To address the research questions, an omnibus 2 (studio audience OAR: Laughter v. Applause) × 3 (partisan affiliation: Democratic v. Republican v. Independent) × 5 (Time) repeated-measures ANOVA was conducted. Because all participants evaluated every studio audience OAR, studio audience OAR and time (i.e., change scores for the 5 s after onset of laughter or applause) served as the within-subjects repeated measure. Political affiliation served as the sole between-subjects variable.

**FIGURE 3:**
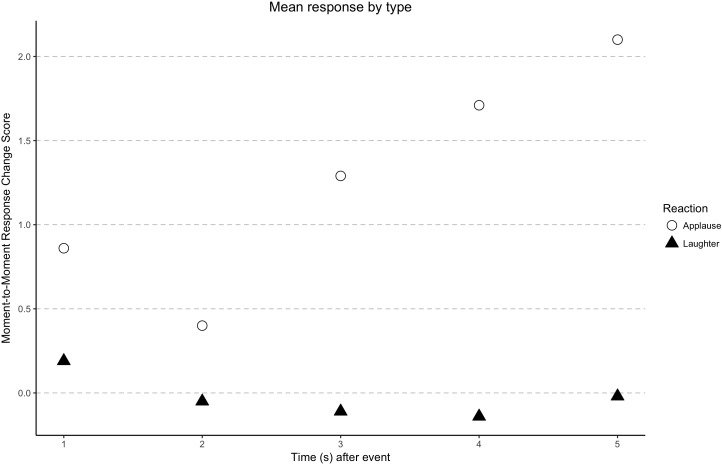
Main effect of studio audience OAR on moment-to-moment response 1–5 seconds after onset.

The main effect of studio audience OAR on MTM response in the continuous response theater was not significance [*F*(2,980) = 3.14, *p* = 0.08, $\eta_{\text{p}}^{2}$ = 0.003]. As seen in **Figure 3**, although studio audience applause elicited more positive MTM response than did laughter, this difference was not statistically significant. This is possibly due to the fact that participants were instructed to give a general evaluation of the speaking candidate, and studio audience OAR was only one of the many factors that influenced real-time candidate evaluation during the presidential debates. Also, this finding suggests that laughter is not necessarily associated with candidate evaluation. While contrary to expectations from Fein et al. (2007), the context is different with strong feelings already held toward the two candidates likely affecting MTM response.

**FIGURE 4:**
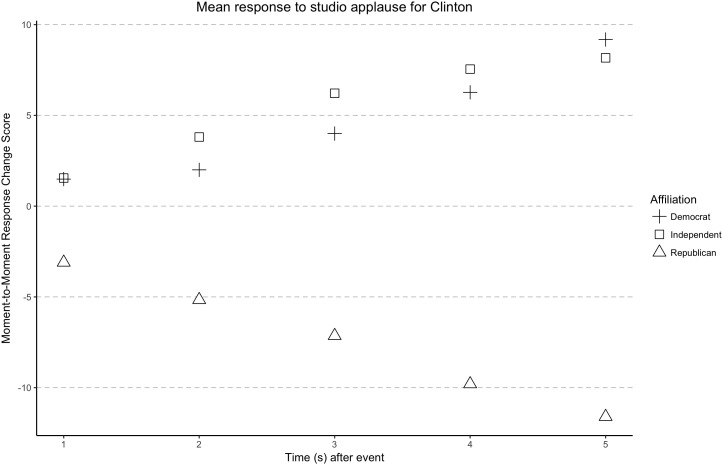
Partisan moment-to-moment response to applause-cheering for Clinton 1–5 seconds after onset.

The main effect of political affiliation on MTM responses was significant [*F*(2,980) = 4.94, *p* = 0.007, $\eta_{\text{p}}^{2}$ = 0.01]. *Post hoc* analysis showed Independent participants’ MTM responses didn’t significantly differ from Democrat (*p* = 0.21) and Republican participants (*p* = 0.15). The significant main effect of political affiliation on MTM responses was primarily driven by the difference between Republican and Democrat participants (*p* = 0.007). To provide a closer examination on the impact of participants’ political affiliation on MTM responses, a series of follow-up analyses were conducted. When the studio audience applauded-cheered, a significant difference in MTM response was found between participants based upon political party affiliation [*F*(2,337) = 3.34, *p* = 0.04, $\eta_{\text{p}}^{2}$ = 0.02]. Specifically, when studio audience applause occurred in response to Clinton’s comments, a significant difference in the continuous response theater participants was found between the three political affiliations [*F*(2,167) = 11.83, *p* < 0.001, $\eta_{\text{p}}^{2}$ = 0.12]. As seen in **Figure 4**, while studio audience applause–cheering elicited more positive MTM responses among Democrat and Independent participants, Republican participants’ MTM responses became more negative when studio audience applauded-cheered for Clinton. Interestingly, no significant difference between participant MTM response based upon political party affiliation when studio audience applause–cheering occurred in reaction to Trump’s comments, *F*(2,167) = 1.56, *p* = 0.21.

After studio audience laughter, participant MTM response didn’t significantly differ between the three political affiliations [*F*(1,643) = 1.22, *p* = 0.30]. These follow-up analyses indicated the main effect of political affiliation on MTM response was primarily due to the difference between Democrat and Republican participants, especially their MTM response to applause instead of laughter. Finally, the studio audience OAR by political affiliation interaction was not significant [*F*(2,980) = 2.38, *p* = 0.09].

### Discussion

Our findings suggest that studio audience applause–cheering had an effect on continuous response study participant candidate evaluations, whereas laughter did not, and that political party affiliation further clarified differences in how likeable the candidates were perceived; however, these findings might not adequately reflect the influence of OAR type. First, this study’s sample was quite small at less than one-third of the comparable studies by Fein et al. (2007) (2 and 3), with statistical power diminished further by small numbers of partisans. Second, our study was carried out during a high stakes election high in a polarized political environment where both candidates were equally likely to win. Finally, and perhaps most important, as can be seen in **Figure 1**, the intensity of studio audience applause–cheering was stronger for most all of their response to both Trump and Clintons’ comments than was that of laughter, making direct comparisons difficult. Fein and colleague’s laboratory studies, while comparable by using continuous response measurement to evaluate response to United States President Ronald Reagan and Minnesota Senator Walter Mondale during their 1984 debates, considered only two studio audience OAR with combined laughter and applause–cheering, and an observable audible and visible reaction from the moderator in one of the instances. Thus, while not as easily parsed as planned laboratory experiments, Study 3 in combination with findings from Study 2, provide real-time evidence of the differential effects of studio OAR on mediated viewers.

## General Discussion

While not as tidy as laboratory experiments, we believe that the enhanced generalizability of our analyses of multiple studies by using ethological methods most proximately building on those pioneered by Robert Provine in his research on laughter (Provine, 2001, 2015) allows for greater and unique insights than provided by other more traditional approaches. Here, we take the position initially promoted by John Wahlke in his 1979 American Political Science Association presidential address and echoed most recently by de Gelder (2017) by asserting that the “prebehavioral” tendencies in social science research, with an emphasis on self-report, miss what the “small data” we use captures (Wahlke, 1979). While we use both approaches throughout our project to triangulate our findings, by focusing on behavioral responses which are more visceral, automatic, and tied to our primate ancestors’ behavior, audible non-verbal utterances such as laughter, applause–cheering, and booing might best reflect behavioral intent of individuals as part of a group.

Observable audience responses such as laughter, applause–cheering, and booing are important because they reflect the emergent properties of individuals becoming groups. While the research reported here does not purport to explain OAR or appraise intent, it makes an important first step in providing evidence concerning individual humans engaging in the group behaviors of applause–cheering, laughter, and (to an extent) booing. In addition to serving the more theoretical purposes of understanding social identity with its evolutionary roots of followership and in-group vs. out-group identities (Haslam et al., 2010; Van Vugt and Ahuja, 2011) and with it the reliability of non-verbal signals (Mehu et al., 2011) inherent in laughter, applause–cheering, and booing (as well as mixtures of these), the research carried out here serves the more proximate and practical needs of understanding the appeal of populist politicians such as Donald Trump, especially in comparison with more traditional candidates. And while we did not systematically explore the booing that occurred, by focusing on the occurrence and effect of laughter and applause–cheering, we have been able to better discriminate between them in terms of form and function.

Findings regarding the specific research questions posited and evaluated in Study 2 suggests that there is a moderately strong relationship between not just the studio audience and the field study audience OAR, answering Research Question 1, but also between laughter occurring in the studio audience and in the field study rooms. When the truncated model was considered, allowing for us to control for studio audience OAR intensity, we found laughter in the studio audience was more strongly related with field study room laughter than applause was with the “all other types” category we used for the field study rooms. This provides evidence responding to Research Question 3. However, while there was modest evidence for Research Question 2, as studio audience OAR intensity was weakly related with field study room cumulative OAR strength, we find, regarding Research Question 4, that there is not a significant relationship between studio audience intensity and OAR type.

The differential response to studio audience OAR was further probed by continuous response measurement (CRM) of MTM liking of the speaking candidate. This allows us to move beyond our research questions to more directly draw inferences. The greater amounts of studio audience laughter elicited by Trump in comparison with Clinton may have affected unaffiliated viewer perceptions by evoking the behavioral mimicry that presumably occurs before social contagion. However, the applause–cheering evoked by Trump may have mattered more, as well as the intensity of the evoked studio audience OAR. Specifically, it appears that the likability of Trump was positively affected by audience applause–cheering to a significantly greater extent than laughter with the CRM study, and that the applause–cheering for Trump was more effective than that elicited by Clinton. In combination with the observational studies regarding the field study, the lack of studio audience control by the moderator may have affected viewer perceptions not just through the stereotypical laughter that is mimicked near automatically, but also by the applause–cheering and mixed audience responses that increase their likability to partisans.

## Future Research

While the information found through the three studies regarding the first general election debate of 2016 helps clarify the role group response in the form of OAR plays, a series of broader questions remain. Specifically, it has been established that individual laughter is a “costly signal” involving abrupt eruptions of distinctive vocalizations concomitant with physiological and emotional change (Grammer and Eibl-Eibesfeldt, 1990; Weisfeld, 1993; Ruch and Ekman, 2001; Gervais and Wilson, 2005; Panksepp, 2007). This might be due to the multi-channel nature of this display; in addition to the vocalic qualities of laughter, distinct facial display signature become evident and co-occur with the laughter (Platow et al., 2005; Mehu and Dunbar, 2008; Mehu, 2011; Stewart et al., 2015). Together, the amusement smiles and audible utterances may be used to differentiate between different types of positive emotional states by how often they occur (Hofmann et al., 2017). Cheering and booing, for their part, both seemingly involve distinctive facial displays co-occurring with the audible utterances. Despite their being more consciously chosen, these types of OAR likely may still lead to change in emotional state and behavioral intent by engaging in two non-verbal channels. However, the influence of applause – which involves only rhythmic hand-and-arm movements – may not necessarily be as reliable an index of individual involvement. At the very least, research should consider more fully the facial display behavior co-occurring with all vocalizations inherent in OAR.

Likewise, questions still remain regarding how individual responses aggregate into a group response. In other words, applause–cheering, laughter, and booing apparently are mimicked, albeit at different levels based upon the audience, and may potentially be socially contagious. As seen in this study, the shared, and potentially mimicked and contagious experience of co-occurring OAR between the studio audience and the field study rooms raises questions. The first, and perhaps foremost, concerns which form of OAR is more likely to lead to group coordination in the form of greater support for goals as stated by the speaker, as well as support for the leader herself or himself. Specifically, while laughter appears to be more likely to be shared than applause–cheering, the nature of booing is not as well established due in great part to its rarity.

At the very least, Studies 1 and 2 suggest a high level of mimicry by individuals, especially regarding laughter. Here, mimicry is defined as the quick and spontaneous matching (within 1 s) of another person’s display behavior and linked with empathy and prosocial behavior (Sachisthal et al., 2016; Moody et al., 2017). Mimicry is thus highly important for social functioning such as group coordination. Social contagion, on the other hand, may be seen as a higher order concept with mimicry being an initial step in an appraisal process whereas individuals assess not just the behavior they are mimicking but also consider their social context (Hatfield et al., 2014). What happened with both the studio and field study audiences with their laughter, however, may reflect mimicry more so than social contagion. This is because social contagion involves appraisal of such factors as social context and group membership (Hatfield et al., 1994, 2014; Lakin et al., 2003), as was seen in the CRM study. On the other hand, field study participants laughing at comments by candidates they did not support (or indeed were predisposed against), could merely be considered mimicry. Whether this ultimately led to social contagion is beyond the purview of this research project; however, it is an important next step in research best considered through more diverse and precise measurement.

A further question concerns whether there are optimal audience sizes for these different forms of OAR; in other words, there tends to be a greater likelihood of applause–cheering, laughter, and booing based upon the increasing size of a group in a form of mutual “grooming” (Dezecache and Dunbar, 2012). However, while evidence suggests that laughter can be a form of mutual grooming amongst two and more individuals (Provine, 2001, 2015) questions remain concerning the numbers of individuals requisite for applause–cheering and booing to occur. Furthermore, there is the question concerning when the group reaches a threshold, will there be a greater likelihood of groups “factioning off” – especially if they are proximate with each other as identifiable entities with separate putative leaders. Furthermore, and related to all the foregoing questions, the mechanism by which individuals are influenced, whether physiological, appraisal-oriented, or emotionally driven group contagion, provides questions to explore in greater detail with a range of different methodologies.

Future research thus should be able to better disambiguate not only the audible signal of group response, but also understand attitudinal and behavioral change. Advances in technology should allow for more precise measurement than that carried out here by naïve judges with limited training. Specifically, audio recorders (including smart phones) placed throughout the room might allow for more accurate notation of OAR timing, type, and intensity, even to the individual level. Indeed, as seen with acoustic research regarding laughter, the different utterances might have a range of signal qualities that are not being considered in needed detail. Just as laughter itself may embody many different emotional messages by reflecting the responses of many different individuals, the resulting message may “get lost in the crowd.” Therefore, by understanding more perfectly the union in OAR such as laughter, applause–cheering, booing, and their combinations, we may be able to divine a greater understanding of the most fundamental of human social activities – politics.

## Notes

Previously presented at the 75th Annual Midwest Political Science Association Conference, April 5–9, 2017, Chicago, IL, United States.

## Ethics Statement

This study (IRB Protocol #: 16-07-029: “The 2016 Presidential Election: Attitudinal Change in Response to Campaign Events, Debates, and Electoral Results”) was carried out in accordance with United States Federal Regulations concerning research [45 CFR 46.102(d)] and human subjects [45 CFR 46.102(f)] as implemented by the University of Arkansas, Fayetteville Office of Research Compliance Institutional Review Board. All participants were given written informed consent in accordance with these United States Federal Regulations and the Declaration of Helsinki.

## Author Contributions

PS: data collection, data cleaning, theory building, writing, and data analysis. AE: data collection, data cleaning, data analysis, and figures. RD: data collection, data cleaning, and editing. ZG: data collection, data cleaning, data analysis, writing, and figures. EB: data collection, data analysis, writing, and editing. RW: data collection and editing. SE: data collection.

## Conflict of Interest Statement

The authors declare that the research was conducted in the absence of any commercial or financial relationships that could be construed as a potential conflict of interest.

## Footnotes

1 These two forms of audience audible utterances are combined for the sake of this analysis; we do appreciate that they reflect different kinds of communication using different non-verbal channels (manipulation of hands and vocalizations) (Schweingruber and McPhail, 1999).
